# Advances in Fetal Repair of Spina Bifida Integrating Prenatal Surgery, Stem Cells, and Biomaterials

**DOI:** 10.3390/biomedicines14010136

**Published:** 2026-01-09

**Authors:** Aleksandra Evangelista, Luigi Ruccolo, Valeria Friuli, Marco Benazzo, Bice Conti, Silvia Pisani

**Affiliations:** 1 Department of Drug Sciences, University of Pavia, Via Torquato Taramelli 12, 27100 Pavia, Italy; aleksandra.evangelista01@universitadipavia.it (A.E.); valeria.friuli@unipv.it (V.F.); bice.conti@unipv.it (B.C.); 2 UOC Othorynolaringology, Fondazione IRCCS Policlinico San Matteo, Via Camillo Golgi 19, 27100 Pavia, Italy; l.ruccolo@smatteo.pv.it (L.R.); marco.benazzo@unipv.it (M.B.); 3 Department of Clinical-Surgical, Diagnostic, and Pediatric Sciences—Integrated Unit of Experimental Surgery, Advanced Microsurgery, and Regenerative Medicine, University of Pavia, Via Ferrata 9, 27100 Pavia, Italy

**Keywords:** spina bifida, myelomeningocele, fetal surgery, prenatal therapy, stem cells, immunomodulation, regenerative medicine, tissue engineering

## Abstract

Spina bifida (SB) is a congenital malformation of the central nervous system (CNS), resulting from incomplete closure of the neural tube (NT) during early embryogenesis. Myelomeningocele (MMC), the most severe form of SB, leads to progressive neurological, orthopedic, and urological dysfunctions due to both NT developmental failure and secondary intrauterine injury (“two-hit hypothesis”). Prenatal repair of MMC has progressed considerably since the Management of Myelomeningocele Study (MOMS, 2011) trial, which showed that open fetal surgery can decrease the need for shunting and improve motor function, although it carries significant maternal risks. To address these limitations, minimally invasive techniques have been developed, with the goal of achieving similar benefits for the fetus while reducing maternal morbidity. Recent research has shifted toward regenerative strategies, integrating mesenchymal stem cells (MSCs), bioengineered scaffolds, and cell-derived products to move beyond mere mechanical protection toward true NT repair. Preclinical studies in rodent and ovine models have shown that amniotic- and placenta-derived MSCs exert neuroprotective and immunomodulatory paracrine effects, promoting angiogenesis, modulating inflammation, and supporting tissue regeneration. Minimally invasive, cell-based interventions such as Transamniotic Stem Cell Therapy (TRASCET), in preclinical rodent models, offer the possibility of very early treatment without hysterotomy, although translation remains limited by the lack of large-animal validation and long-term safety data. In parallel, advances in biomaterials, nanostructured scaffolds, and exosome-based therapies reinforce a regenerative paradigm that may improve neurological outcomes and quality of life in affected children. Ongoing translational studies are essential to optimize these approaches and define their safety and efficacy in clinical settings. This review provides an integrated overview of embryological mechanisms, diagnostic strategies, and prenatal therapeutic advances in SB treatment, with emphasis on prenatal repair, fetal surgery and emerging regenerative approaches.

## 1. Introduction

Spina bifida (SB) is a rare congenital malformation of the central nervous system (CNS), representing one of the most frequent fetal congenital anomalies compatible with life. It arises from the incomplete closure of the neural tube (NT) during early embryonic development, typically between the third and fourth weeks of gestation [1]. This failure in NT closure leads to structural defects of the vertebral column, most frequently involving absence or malformation of the posterior vertebral arches, leading to incomplete formation of the spinal canal [2].

The most severe form, myelomeningocele (MMC), has an estimated global prevalence of approximately 35–50 cases per 100,000 live births, although this figure shows substantial regional variability according to folic acid fortification policies and surveillance methods [3].

According to the systematic meta-analysis by Atta et al., the pooled prevalence of spina bifida was 33.9 per 100,000 live births in regions with mandatory folic acid fortification—predominantly North and South America, Canada, and Oceania—compared with 48.4 per 100,000 live births in regions with voluntary or absent fortification, such as most European and Asian countries. When studies also included stillbirths and pregnancy terminations, the prevalence was 35.2 per 100,000 births in countries with mandatory fortification versus 52.3 per 100,000 in those without. Geographically, the lowest prevalence was reported in North America (≈38.7 per 100,000 live births), while Asia and parts of Africa showed the highest estimates, occasionally exceeding 80–200 per 100,000, reflecting limited access to supplementation and variable registry coverage. Despite widespread public health campaigns, SB persists even in countries with established preventive programs, highlighting persistent inequalities in nutrition, healthcare access, and prenatal education.

Beyond its clinical complexity, MMC imposes a substantial socio-economic burden on individuals, families, and healthcare systems. Patients often require lifelong multidisciplinary care, with repeated surgical procedures and continuous neurological, orthopedic, and urological follow-up. Physical rehabilitation and neuropsychological support are also frequently necessary. Functional complications, such as lower limb paralysis, skeletal deformities, recurrent urinary tract infections, and dependence on mobility aids, translate into high direct medical costs as well as indirect costs related to loss of autonomy, caregiver burden, and reduced quality of life (QoL). These challenges are further exacerbated in low-resource settings, where limited access to prenatal screening, folic acid supplementation, and adequate postnatal care contributes to worse outcomes and lower survival rates [4].

Early prenatal diagnosis is therefore crucial to mitigate the socio-economic impact of SB, enabling timely decision-making and access to prenatal therapeutic options. Despite decades of preventive efforts, spina bifida remains a major global public health issue, with its prevalence and burden closely tied to the implementation and effectiveness of prevention strategies. Importantly, the lifetime cost of care for an individual with spina bifida is estimated to exceed 500,000–1,000,000 USD, reflecting the substantial long-term healthcare, surgical, orthopedic, neurological, and urological support required [5].

This review aims to provide a comprehensive overview of SB, with particular focus on its prenatal treatment and management. It begins with the anatomical and pathophysiological basis of the condition, exploring the classification of SB and the embryological mechanisms underlying neural tube defects (NTDs). Special attention is given to the clinical implications affecting the nervous, orthopedic, and urological systems.

Current strategies for early diagnosis are then examined, including imaging modalities and biochemical markers, with emphasis on the importance of timely prenatal detection. A central section addresses prenatal treatment approaches, comparing open fetal surgery and fetoscopic techniques in terms of safety, efficacy, and maternal–fetal outcomes. The discussion also considers complementary pharmacological and nutritional interventions, as well as the status of ongoing clinical trials. Emerging regenerative therapies, including stem cell applications, biomaterials, organoids, and molecular targets, are discussed, highlighting both their potential and the current limitations of clinical translation [6].

Finally, the review critically examines the existing challenges—technical, ethical, and regulatory—and outlines future directions such as the need for large-scale clinical studies, global registries, and the integration of advanced technologies like gene therapy and nanomedicine. Through this analysis, the goal is to inform and advance prenatal clinical practice while identifying priority areas for future research in the management of SB.

### 1.1. Anatomy and Pathophysiology

**Figure 1:**
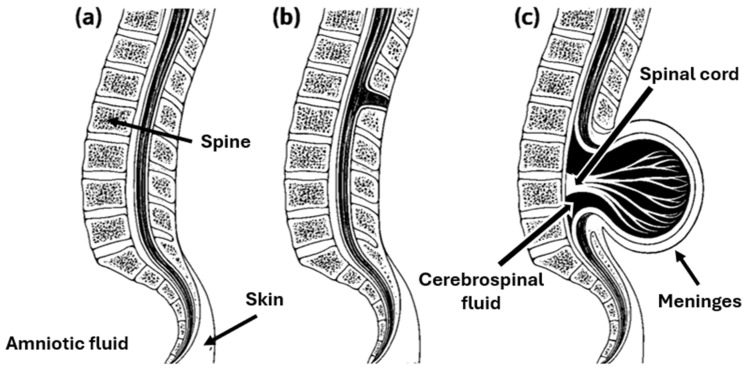
Morphological classification of spina bifida, illustrating (**a**) regular spinal anatomy, (**b**) the closed form (spina bifida occulta) characterized by a vertebral arch defect without exposure of neural tissue, and (**c**) the open form (MMC), showing herniation of the spinal cord and meninges through the vertebral defect.

SB encompasses a spectrum of neural tube defects (NTDs), morphologically classified as closed and open forms depending on the degree of neural tissue exposure (Figure 1). This distinction carries important prognostic value and guides both prenatal and postnatal surgical planning. Closed spina bifida (CSB), or spina bifida occulta, is confined to a defect of the vertebral arch and is completely covered by skin. It is often asymptomatic and may remain undiagnosed until adolescence or adulthood, though in some cases it can be associated with tethered cord syndrome or subtle neurological manifestations. Open spina bifida (OSB) includes the more severe phenotypes, particularly meningocele and MMC. In meningocele, the meninges herniate through a vertebral defect to form a cerebrospinal fluid-filled sac, while the spinal cord itself remains intact and typically unaffected. By contrast, MMC, the most common and clinically significant form, involves herniation of both the spinal cord and meninges through a vertebral defect into a sac that lacks skin coverage. This open lesion directly exposes neural tissue to the intrauterine environment, where it is vulnerable to mechanical trauma and the neurotoxic effects of amniotic fluid [7]. The pathophysiology of MMC is explained by the “two-hit hypothesis”, whereby an initial failure of neural tube closure is compounded by ongoing intrauterine injury to the malformed and exposed spinal cord. In the absence of timely intervention, this process leads to flaccid paralysis of the lower limbs, bladder and bowel dysfunction, and frequent association with Chiari II malformation, which may result in hydrocephalus. While hydrocephalus has traditionally been managed with ventriculoperitoneal (VP) shunt placement, current practice in many centers increasingly favors endoscopic cerebrospinal fluid diversion strategies, such as endoscopic third ventriculostomy (ETV) with or without choroid plexus cauterization (ETV/CPC), either as first-line treatment or as an alternative to shunting [8].

A morphological variant of MMC, termed myeloschisis, is characterized by a completely open neural plate lying flat against the skin surface without dermal or meningeal covering. This configuration results in uninterrupted exposure of neural tissue throughout gestation, with consequent profound neurological damage. Despite advances in fetal and neonatal care, neonatal mortality in MMC remains approximately 10%, and among survivors only about half achieve functional independence in adulthood. Current management options include termination of pregnancy, postnatal neurosurgical repair, or prenatal (fetal) surgical correction, the latter aiming to limit secondary neural injury and improve long-term outcomes [9].

**Figure 2:**
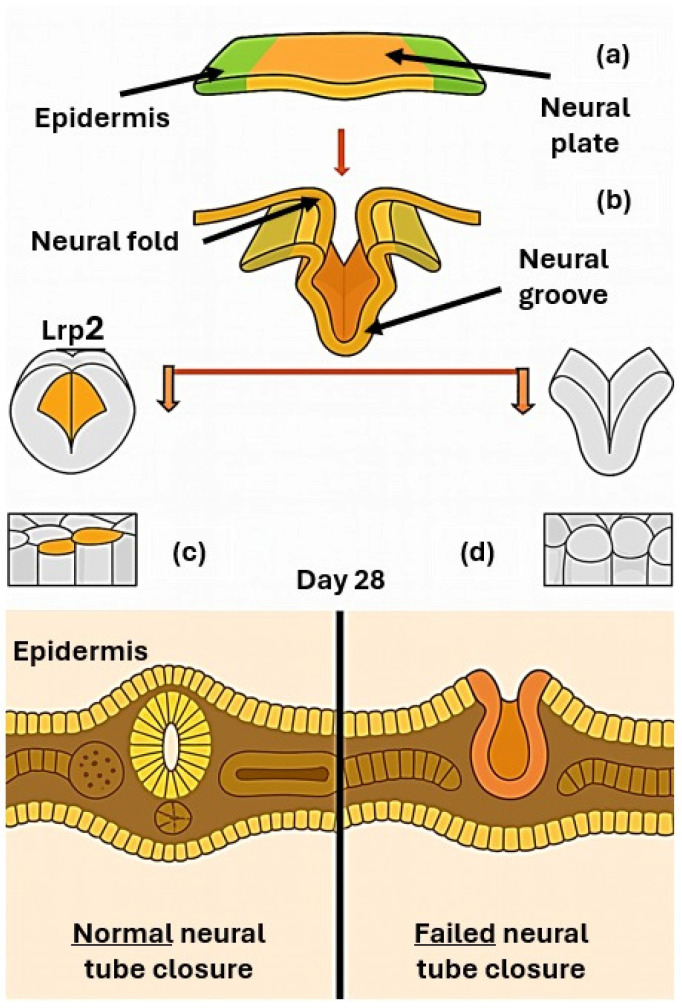
Scheme of neurulation developmental process showing: (**a**) the flat neural plate (**b**) its bending and fusion to generate the neural tube, the precursor of the central nervous system, (**c**) regular neural tube closure, (**d**) failed neural tube closure.

MMC originates from a failure of primary neurulation, a highly regulated morphogenetic process occurring between days 22 and 28 of gestation. During this stage, the neural plate elevates, bends, and fuses along the midline to form the neural tube, the precursor of the central nervous system (Figure 2). Closure of the cranial and caudal neuropores is normally completed by day 25 and day 28, respectively; failure of posterior neuropore closure results in open NTDs such as MMC [4]. The proper completion of neurulation depends on the interplay of genetic, molecular, and biomechanical mechanisms, including precise gene regulation, cell shape changes, planar polarity signaling, and adequate folate metabolism. Genetic mutations, epigenetic alterations, or environmental influences can disrupt these processes, leading to persistent vertebral defects and exposure of the spinal cord and meninges through the open lesion [6]. The pathogenesis of MMC is classically explained by the two-hit hypothesis, in which the first insult corresponds to embryologic failure of neural tube closure, while the second hit reflects progressive intrauterine damage to the exposed neural tissue. This ongoing injury—driven by mechanical trauma, inflammation, and the cytotoxic effects of amniotic fluid—results in axonal loss, gliosis, and structural disorganization of the spinal cord, as confirmed by histopathological studies. These mechanisms collectively underline the neurological deterioration characteristic of MMC and provide the rationale for early prenatal detection and surgical repair aimed at preserving neural function [10].

At the molecular level, neural tube closure depends on tightly regulated mechanisms such as apical constriction, neuroepithelial bending, and midline fusion of the neural folds. The endocytic receptor Lrp2 (megalin) has emerged as a pivotal player in this context. Studies in mouse and Xenopus laevis demonstrate that loss of Lrp2 profoundly disrupts neuroepithelial morphogenesis, preventing proper folding and closure. Lrp2 is highly expressed at the apical surface of neuroepithelial cells, where it mediates the removal of excess apical membrane during apical constriction. Its absence leads to defective hinge point formation, impaired elevation of neural folds, and abnormal localization of the planar cell polarity (PCP) protein Vangl2, which is essential for convergent extension. Disrupted Vangl2 trafficking results in loss of planar polarity and defective directional cell behavior, further compromising neural tube closure [11]. Beyond Lrp2-dependent regulation, additional pathways such as Wingless-Related Integration Site Signaling Pathway (Wnt) signaling, cytoskeletal dynamics, and transcriptional control are crucial for neural fold convergence and fusion. Disruption of these networks impairs the convergence and fusion of neural folds, leading to open spina bifida. The two-hit hypothesis remains a unifying framework, emphasizing that MMC is not only the consequence of a developmental failure but also of progressive intrauterine damage, which together shape its clinical severity [12]. Experimental studies in Lrp2-deficient animal models have provided further insight into the mechanisms underlying neural tube defects. These models demonstrate two pathological trajectories: dilation of the dorsal neural tube leading to midline anomalies (such as choroid plexus malformations and skull defects), and failure of anterior neuropore closure resulting in atypical anencephaly. Both are accompanied by posterior spinal defects due to incomplete neural fold elevation and fusion. Neuroepithelial cells show enlarged apical surfaces and fail to form a proper floor plate, while disrupted planar cell polarity further impairs closure. Together, these findings suggest that spina bifida in Lrp2–/– embryos arises from combined mechanical and signaling defects [11].

The neurological outcome depends largely on the anatomical level of the lesion: higher lesions correlate with more severe motor and sensory impairment of the lower limbs. Clinically, MMC is associated with flaccid paralysis, sensory loss, neurogenic bladder and bowel dysfunction, and orthopedic deformities such as clubfoot and scoliosis. Chronic intrauterine exposure also disrupts the structural organization of the spinal cord, limiting the potential for functional recovery even when prenatal intervention is performed [13]. Chiari II malformation is almost universally associated with MMC and involves herniation of the cerebellar tonsils and brainstem through the foramen magnum. This anatomical distortion alters cerebrospinal fluid dynamics, contributing to hydrocephalus, cranial nerve dysfunction, and brainstem compression. Although hydrocephalus has historically been managed with early ventriculoperitoneal (VP) shunt placement, contemporary management in many centers increasingly includes endoscopic cerebrospinal fluid diversion techniques, such as endoscopic third ventriculostomy (ETV) with or without choroid plexus cauterization (ETV/CPC), which may reduce long-term shunt dependence. Before the NIH-sponsored MOMS trial, preliminary evidence had suggested that prenatal surgical repair could partially reverse hindbrain herniation, lower the incidence of shunt-dependent hydrocephalus, and improve motor outcomes in selected patients [14]. Histological examinations of exposed spinal cords in MMC confirm ongoing tissue degeneration, with disorganization of gray and white matter, incomplete meningeal and vertebral arch formation, and herniation of the neural placode into a cerebrospinal fluid-filled sac. Chronic exposure induces fibrosis, inflammatory infiltration, and loss of neural pathways, ultimately compromising neuromotor development [12]. Beyond its embryological basis, MMC leads to multisystem consequences that shape long-term QoL. The extent of neurological, orthopedic, and urological complications is primarily determined by the anatomical level and size of the spinal defect.

#### Multisystem Clinical Manifestations of MMC

**Figure 3:**
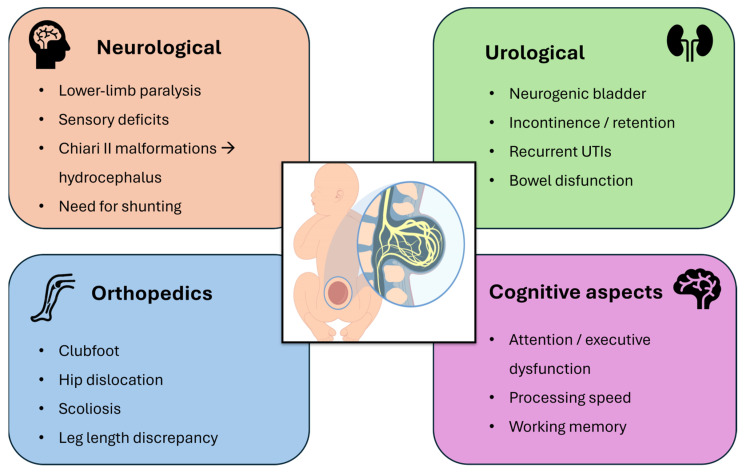
Schematic overview of the multisystem clinical involvement in myelomeningocele, highlighting key neurological, urological, orthopedic, and cognitive deficits.

MMC produces a wide range of multisystem clinical consequences involving neurological, urological, orthopedic, and cognitive domains (Figure 3). Neurologically, lesions typically located in the lumbar and sacral regions lead to flaccid paralysis or weakness of the lower limbs, reduced or absent deep tendon reflexes, and dermatomal sensory loss. These deficits arise from the combined effects of primary neural tube closure failure and secondary neurodegeneration driven by chronic exposure of the spinal cord to inflammatory and proteolytic components of the amniotic fluid. Chiari II malformation is present in the vast majority of cases, with cerebellar and brainstem herniation impairing cerebrospinal fluid circulation and contributing to hydrocephalus, which often necessitates early ventriculoperitoneal shunting [15].

Autonomic involvement results in neurogenic bladder dysfunction, manifested by urinary retention, incontinence, and recurrent urinary tract infections. Chronic bowel dysfunction—ranging from constipation to fecal incontinence—is also common, and only a minority of patients achieve spontaneous continence despite long-term management strategies such as clean intermittent catheterization, anticholinergic therapy, or surgical interventions [16].

Orthopedic complications stem from impaired neuromuscular control and reduced fetal mobility, predisposing affected infants to talipes equinovarus (clubfoot), hip dislocation, scoliosis, and joint contractures. Additional musculoskeletal abnormalities, including tibial torsion, kyphosis, and leg-length discrepancy, are frequently observed. Data from the MOMS trial indicate that prenatal repair may reduce the incidence of leg-length discrepancy at early follow-up, suggesting secondary benefits for musculoskeletal development [17].

Finally, children with MMC often experience cognitive difficulties, including impairments in attention, executive function, processing speed, and working memory. These challenges can significantly affect academic performance and psychosocial development, even in the presence of optimized motor outcomes [18].

Overall, the neurological, orthopedic, and urological sequelae of MMC are profound and multifaceted. These multisystem deficits underscore the need for lifelong, coordinated multidisciplinary management to optimize function and QoL.

### 1.2. Diagnosis and Screening

Early and accurate prenatal diagnosis of SB is essential for informed decision-making and optimized perinatal care. The condition is usually detected during the second-trimester anomaly scan (18–26 weeks), with high-resolution ultrasound serving as the gold standard first-line modality. Ultrasound allows identification of both direct and indirect signs of open neural tube defects. Direct signs include visualization of a fluid-filled sac protruding from the spinal canal, typically at lumbar or sacral levels, together with vertebral anomalies. Indirect cranial markers reflect the downward displacement of posterior fossa structures characteristic of Chiari II malformation. The most recognized are the “lemon sign,” denoting bilateral frontal bone scalloping, and the “banana sign,” describing abnormal curvature and downward displacement of the cerebellum. Additional findings may include ventriculomegaly, obliteration of the cisterna magna, and limb deformities such as clubfoot, which suggest lower motor neuron involvement [19]. When ultrasound findings are suggestive but inconclusive, fetal magnetic resonance imaging (MRI) provides valuable complementary information. MRI offers superior soft tissue contrast and detailed evaluation of intracranial anatomy, including posterior fossa structures, brainstem displacement, and corpus callosum integrity. These data are particularly relevant for surgical planning and for assessing eligibility for prenatal repair, which is usually performed in the late second or third trimester [6].

Amniocentesis may be selectively used to measure alpha-fetoprotein (AFP) and acetylcholinesterase (AChE) levels, both typically elevated in OSB. Maternal serum AFP (MSAFP) screening, performed between 15 and 18 weeks, remains a widely used non-invasive tool; values > 2.5 multiples of the median (MoM) are strongly suggestive of an open defect. When combined with targeted ultrasound, AFP screening improves sensitivity and facilitates timely referral for fetal therapy evaluation. Genetic analysis of amniotic fluid may also help exclude chromosomal abnormalities or syndromic conditions, though invasive testing is not routinely indicated in isolated MMC without additional anomalies [20]. Ongoing research aims to establish first-trimester diagnostic markers, such as the “crash sign” and abnormal intracranial translucency. While promising, these markers still require validation before they can be integrated into routine practice [21]. The timing of diagnosis plays a pivotal role in determining clinical outcomes. Early detection enables referral to specialized fetal surgery centers, optimized delivery planning, and comprehensive parental counseling regarding prognosis and treatment options. For fetuses eligible for prenatal repair, identification before irreversible spinal cord injury occurs can significantly improve lower-limb motor function and reduce the risk of hindbrain herniation and hydrocephalus [19]. Ultimately, timely diagnosis not only influences therapeutic eligibility but also shapes long-term neurological and developmental trajectories, underscoring its central role in modern fetal medicine.

## 2. Prenatal Treatments

Surgical repair remains the cornerstone of SB management and can be performed either after birth (postnatally) or during gestation (prenatally). Comparative studies, including the landmark MOMS trial and subsequent series, have demonstrated the superiority of prenatal surgery in several fetal outcomes. In particular, in utero repair significantly reduces the need for cerebrospinal fluid shunting, promotes partial or complete reversal of Chiari II malformation, and improves motor function and ambulatory potential compared with postnatal closure [22]. Orthopedic benefits have also been observed, with better lower-limb alignment and fewer secondary deformities. Nevertheless, prenatal intervention entails greater maternal risk, including uterine dehiscence, preterm delivery, and postoperative morbidity, and requires specialized multidisciplinary teams available only in referral centers. Postnatal repair, on the other hand, remains the standard approach worldwide, owing to its broader accessibility, predictable perioperative course, and lower maternal burden. Overall, prenatal surgery offers superior neurological and functional outcomes for the child, at the cost of increased maternal risk and logistical complexity [17]. The following sections discuss in detail the two main prenatal approaches, open fetal surgery and fetoscopy repair, highlighting their respective indications, benefits, and limitations.

Open fetal surgery is the most established prenatal treatment for MMC, first performed via hysterotomy in 1997. By 2003, over 200 procedures had been reported, with marked reductions in hindbrain herniation compared with historical controls; however, early experience was tempered by fetal/neonatal mortality, prematurity, and notable maternal morbidity [1]. The classic approach involves maternal laparotomy and uterine hysterotomy to allow direct access for repositioning of neural tissue and multilayer closure (dura, muscle, skin). Procedures are performed under general anesthesia with uterine relaxation and continuous fetal monitoring; the uterus is closed watertight to limit membrane rupture. The MOMS standardized inclusion criteria (19–26 weeks; isolated MMC; normal karyotype) and showed significant advantages of prenatal over postnatal repair, lower VP shunting (44% vs. 83.7%), reduced Chiari II malformation, and higher independent ambulation at 30 months (44.8% vs. 23.9%) [23]. Despite these benefits, maternal and obstetric risks remain substantial. Preterm premature rupture of membranes is common; most patients deliver preterm; and uterine scar complications can occur, with implications for subsequent pregnancies and delivery planning. Cesarean delivery is generally required for the index and future pregnancies. Furthermore, even after prenatal repair, a considerable proportion of children do not achieve independent ambulation at 30 months, indicating that surgical closure alone cannot fully restore motor function [24].

Open fetal surgery remains the reference standard for prenatal MMC repair and is performed via maternal laparotomy and hysterotomy, allowing direct multilayer closure of the fetal defect. The MOMS trial established the criteria for patient selection (19–26 weeks of gestation; isolated T1–S1 lesion with hindbrain herniation; absence of additional anomalies) and demonstrated clear advantages over postnatal correction—namely, reduced ventriculoperitoneal shunt placement (≈40% vs. 82%), improved independent ambulation at 30 months, and attenuation of Chiari II malformation [25]. These findings provided the biological rationale for early in utero intervention aimed at preventing secondary neural injury caused by prolonged amniotic exposure. Despite these fetal benefits, open surgery carries substantial maternal–obstetric risk. In MOMS, mean gestational age at delivery was lower in the prenatal group, with higher rates of preterm premature rupture of membranes (PPROM), uterine dehiscence, and the need for cesarean delivery in current and subsequent pregnancies [6]. Consequently, strict patient selection, specialized surgical expertise, and long-term multidisciplinary follow-up are essential to ensure maternal safety. Recent preclinical work seeks to enhance neurological outcomes through regenerative adjuncts. In ovine models, umbilical cord-derived MSCs (UC-MSCs) applied at the repair site improved early motor function, preserved neuronal tissue, and reduced fibrosis through paracrine signaling [26]. Similarly, alginate microparticles delivering basic fibroblast growth factor (bFGF) promoted defect coverage and epithelialization in experimental MMC [9]. These findings suggest that the combination of surgical repair with biologically active materials may evolve fetal surgery from a merely protective procedure toward a regenerative intervention.

**Figure 4:**
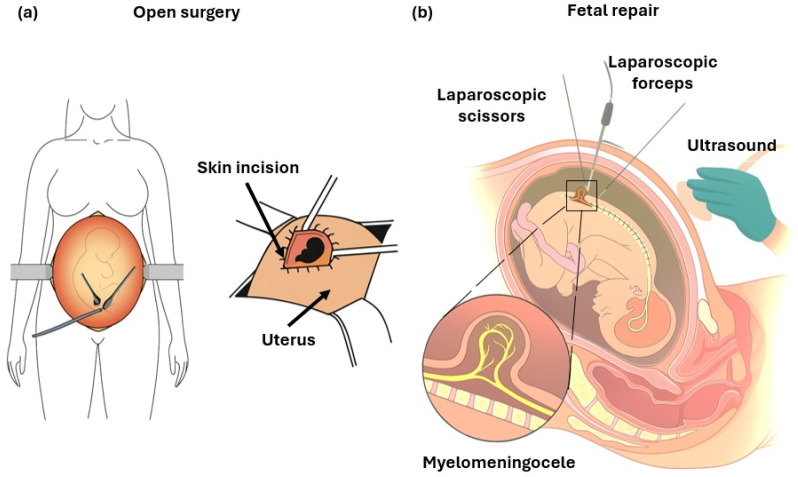
Schemes of: (**a**) Open fetal surgery: after maternal laparotomy and hysterotomy, the uterus is exteriorized to allow direct multilayer closure of the fetal defect under visualization. (**b**) Fetoscopic repair: minimally invasive technique performed under ultrasound guidance through small uterine ports, using laparoscopic instruments (scissors and forceps) to close the defect. The insert illustrates the exposed neural placode characteristic of MMC.

Two main approaches are currently used for prenatal surgical repair of myelomeningocele: open fetal surgery and fetoscopy repair (Figure 4). To reduce these risks while maintaining fetal benefit, several fetoscopy techniques have been developed. These include percutaneous fetoscopy, performed through ultrasound-guided trocar insertion, and laparotomy-assisted (hybrid) approaches, in which small uterine ports are introduced via a limited maternal incision. Across centers, the main technical elements that vary include the access strategy (fully percutaneous versus hybrid), the use and parameters of CO_2_ insufflation (pressure, flow, and duration), and the method of defect closure (primary suturing versus patch-assisted repair). In both approaches, the spinal placode is repositioned and the defect is closed endoscopically using sutures and/or patches, with CO_2_ insufflation employed to optimize visualization of the surgical field [6]. Data from the International Fetoscopic Myelomeningocele Repair Consortium, comprising more than 300 procedures, indicate substantially lower maternal complication rates compared with open repair—specifically, absence of uterine dehiscence, reduced postoperative pain, shorter hospitalization, and the possibility of vaginal delivery in up to one-third of cases [27]. Nonetheless, fetoscopic repair remains technically challenging, is associated with longer operative times, and continues to show higher rates of PPROM relative to open repair. Despite these limitations, fetoscopy represents a significant advancement toward minimally invasive fetal surgery, seeking to preserve the neurological benefits of early prenatal closure while minimizing maternal risk [28]. The following section discusses in detail the outcomes and current refinements of these two surgical strategies.

Several challenges persist, including technical complexity, longer operative times, a steep learning curve, and high PPROM rates reported in some series. Inter-center variability and the absence of large, randomized trials directly comparing fetoscopic and open techniques limit firm conclusions. Moreover, long-term outcomes—such as ambulation, continence, cognition, and tethered cord risk, potentially influenced by skin-over-biocellulose closures—require standardized follow-up and reporting [29].

The percutaneous–mini-laparotomy (PML) hybrid reported, in a preliminary series of seven cases, mean delivery at 36.1 weeks, PPROM in 57%, no major maternal morbidity, and 71% vaginal delivery, potentially avoiding obligatory cesarean in future pregnancies. Larger prospective cohorts are needed to determine whether PML can match or surpass open repair for neurodevelopmental outcomes, delivery timing, and maternal safety; nonetheless, it represents a meaningful evolution in prenatal MMC surgery [30]. More than a decade after MOMS, prenatal MMC repair has reshaped clinical practice, though adoption remains uneven. Open fetal surgery (per MOMS) is the current benchmark, reducing cerebrospinal fluid (CSF) shunting, improving motor outcomes, and decreasing hindbrain herniation [22]. Expanded analyses confirmed benefits: among 183 patients, 12-month shunt placement was 44% vs. 84% with postnatal repair, with fewer shunt revisions (15.4% vs. 40.2%) [31]. Observational data suggest that high-volume centers (≥30 cases) achieve fewer complications, lower prematurity rates, and better neurological outcomes.

Several barriers persist, including technical complexity, the need for specialized multidisciplinary teams, and obstetric consequences such as the requirement for cesarean delivery in future pregnancies. While fetoscopy approaches reduce maternal morbidity—for example, by eliminating the risk of uterine dehiscence—they may also be associated with high rates of PPROM and variable shunt requirements across published series [22]. Heterogeneity in technique, outcome definitions (e.g., evolving shunt criteria during MOMS), and institutional resources complicates comparisons. Critically, the absence of large phase II/III randomized trials under standardized protocols prevents a unified international consensus.

Looking ahead, research targets refinements in timing (e.g., <20 weeks) and neuroprotective alternatives such as endoscopic third ventriculostomy (ETV) with choroid plexus cauterization (CPC) to further decrease shunt dependency [31]. In parallel, early-phase trials are testing MSC adjuncts (placental/umbilical) to fetal surgery, leveraging neuroprotective, anti-inflammatory, and anti-fibrotic effects; ovine models showed improved motor neuron survival, reduced fibrosis, and enhanced locomotion, now informing phase I/II feasibility studies [12]. Combining MSCs with biomaterial scaffolds delivered in utero may shift fetal surgery from a purely protective to a regenerative paradigm.

Open prenatal repair remains the benchmark for eligible cases. Fetoscopic and biologically advanced strategies are promising but require validation in multicenter phase II/III trials with standardized criteria. Until then, prenatal MMC repair will remain concentrated in high-expertise centers rather than a universally accessible standard of care.

## 3. Emerging Therapies: Stem Cells and Biomaterials

Recent advances in prenatal medicine have prompted the development of innovative strategies aimed at protecting the exposed neural tissue and enhancing functional recovery following myelomeningocele repair. Beyond conventional surgical closure, these emerging approaches seek to mitigate secondary neurodegeneration, promote regeneration, and ultimately improve postnatal neurological and functional outcomes.

To this end, multidisciplinary research has focused on regenerative and bioengineering solutions, including stem cell-based therapies, biomaterial scaffolds, and molecular or pharmacological adjuncts. These efforts aim to transform fetal repair from a purely protective intervention into a biologically restorative treatment capable of supporting long-term neurodevelopmental benefit.

Growing interest has therefore turned toward regenerative adjuncts designed to enhance fetal repair. MSCs, which exhibit neuroprotective and anti-inflammatory properties, together with bioengineered scaffolds, have shown promising results in ovine and rodent models [32]. Early clinical-grade applications of placental MSCs delivered as an adjunct to surgical repair have reported encouraging functional signals, supporting further translational research and future clinical trials. Persistent maternal risk and the incomplete functional recovery observed after standard fetal surgery underscore the rationale for developing such regenerative strategies.

### 3.1. Stem Cell Approaches

Cell-based, non-surgical strategies are emerging as a promising frontier in prenatal therapy, aiming to promote neural protection and epithelial regeneration without the need for invasive surgery. However, it should be emphasized that the current evidence for these approaches, particularly TRASCET, remains largely preclinical and limited to rodent models, with important challenges related to cell tracking, functional outcome assessment, and translational scalability. Among these, TRASCET has shown particularly encouraging results. This technique involves ultrasound-guided intra-amniotic injection of MSCs derived from amniotic fluid MSCs (AF-MSCs) or placental tissue MSCs (P-MSCs), delivered during early gestation [33].

Preclinical studies in fetal mouse models demonstrated partial to complete epithelialization of the exposed spinal cord, suggesting that transplanted MSCs can migrate toward the lesion site, secrete neurotrophic and angiogenic factors, and facilitate spontaneous coverage of the defect. These findings support the concept of early gestational regenerative intervention, in which cellular therapy could complement or precede conventional surgical repair to enhance neural preservation and improve long-term functional outcomes.

MSCs are promising for prenatal MMC owing to regenerative, immunomodulatory, and neuroprotective actions that are largely paracrine, with secretion of trophic factors supporting neural survival, tempering inflammation, and enhancing repair [34]. In this context, immunomodulation plays a central role in mitigating the inflammatory microenvironment associated with secondary neural injury in spina bifida, positioning MSC-based interventions within an emerging framework of immunoregenerative therapy. AF-MSCs and PMSCs have enabled minimally invasive strategies such as TRASCET. In fetal mice, intra-amniotic injection of P-MSCs or AF-MSCs promoted epithelial coverage of the defect via paracrine effects [35]. Watanabe et al. showed spontaneous adhesion/proliferation of amniotic mesenchymal-like cells on gelatin scaffolds, implicating endogenous fetal progenitors in repair [10]. P-MSCs exhibit strong neuroprotective/anti-apoptotic effects without engraftment, fostering neurite outgrowth and improved cord integrity in models [36]. Umbilical cord MSCs (UC-MSCs)– and Wharton’s Jelly-derived MSCs (WJ-MSCs) also show encouraging signals. In an ovine fetal-surgery model, topical allogeneic UC-MSCs improved motor function, increased neuronal density, reduced fibrosis, and restored urinary continence without tumor formation/systemic migration [26]. In pediatrics, intravenous WJ-MSCs were well tolerated and correlated with improvements in urinary/bowel control, cognition, and QoL, although randomized trials are still needed. Bone marrow-derived MSCs (BM-MSCs) combined with scaffolds have demonstrated survival, migration, and neural differentiation in fetal rats, facilitating coverage and regeneration [37]. Gene-enhanced BM-MSCs overexpressing brain-derived neurotrophic factor (BDNF) improved engraftment, neuronal preservation, and reduced apoptosis; intrathecal BM-MSCs showed targeted migration toward sensory pathways with partial differentiation into Brn3a-positive sensory neurons [38]. MSC-based prenatal/perinatal therapies are feasible and biologically compatible for MMC. Their paracrine neuroprotective effects, immunomodulatory capacity, and alignment with immunoregenerative therapeutic principles, together with low immunogenicity and scaffold compatibility, support ongoing translational efforts; key priorities include optimization of delivery route, timing, and dose, as well as establishment of long-term safety and efficacy [39].

### 3.2. Biomaterials and Scaffolds

Tissue engineering represents a rapidly evolving adjunct to fetal surgery, aiming not only to provide mechanical protection but also to create bioactive environments that promote neural regeneration, angiogenesis, and epithelial closure in utero. Modern scaffold design combines biomimetic architecture, cellular integration, and controlled release of trophic factors to reproduce key aspects of fetal tissue healing.

Natural and hydrogel-based materials have shown remarkable promise for minimally invasive applications. In fetal rat models, gelatin-based sponges and microspheres promoted superior epidermal ingrowth and cell adhesion compared with planar sheets, largely due to their enhanced porosity and surface area. Injectable gelatin microspheres have proven compatible with ultrasound-guided intra-amniotic delivery, enabling treatment as early as 14–15 weeks of gestation [10]. Similarly, chitosan-gelatin composites seeded with BM-MSCs facilitated epithelial coverage and axonal repair through an interconnected porous matrix [37]. In fetal sheep, a fetal dermo-epidermal skin substitute (fDESS) composed of autologous keratinocytes and fibroblasts cultured on a collagen hydrogel integrated efficiently into fetal wounds, forming a mature epidermal layer within four weeks [40].

Among rigid and collagen-based scaffolds, type I collagen hydrogels seeded with fetal chondrocytes demonstrated excellent chondrogenic differentiation and structural stability, underscoring the importance of scaffold stiffness and biochemical composition for neural protection [41]. Heparin-functionalized collagen matrices incorporating growth factors such as fibroblast growth factor 2 (FGF2) and vascular endothelial growth factor (VEGF) enhanced epithelialization and reduced fibrotic remodeling in fetal sheep, supporting their role in scarless fetal healing [42].

Biological membranes and MSC-delivery platforms have emerged as versatile alternatives for combining structural support with paracrine signaling. In fetal rat models, cryopreserved human umbilical cord (HUC) membranes promoted angiogenesis and multilayer tissue formation with minimal inflammation, outperforming biocellulose films (BCF) owing to their extracellular matrix components (HC-HA/PTX3) [43]. Likewise, fibrin-based patches delivering UC-MSCs in fetal sheep achieved uniform coverage, reduced fibrosis, and decreased tethering of the repaired spinal cord [44]. Commercial collagen dural substitutes (DuraGen^®^, Durepair™—Integra LifeSciences Holdings Corp., Princeton, NJ, USA) also preserved ventral horn morphology and mitigated glial activation in fetal rabbits, providing valuable benchmarks for translational development [33].

At the micro- and nanoscale, nanostructured and electrospun scaffolds offer precise architectural cues for guided neuronal growth. Aligned electrospun poly-l-lactic acid (PLLA) nanofibers, mimicking the natural extracellular matrix, provided directional axonal guidance and successful integration without fibrosis in large-animal models. Hybrid constructs combining semicircular inner and rectangular outer nanoscaffolds demonstrated prenatal feasibility and supported organized neuronal remodeling [45].

More recently, synthetic elastomers such as poly(1,8-octanediol-co-citrate) (POC) and small intestinal submucosa (SIS) matrices have shown elasticity, resistance to contraction, and reduced fibrosis compared to unseeded controls [39]. When pre-vascularized with induced pluripotent stem cell-derived endothelial cells, these constructs improved oxygenation, vascular integration, and paracrine support to adjacent neural and urothelial tissues.

**Figure 5:**
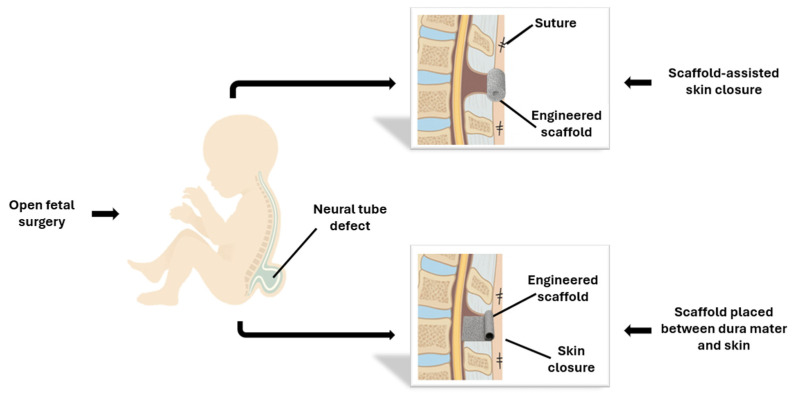
Fetal repair of MMC using tissue-engineered scaffolds. In the open fetal surgery approach, performed through maternal laparotomy and hysterotomy, an engineered scaffold is positioned over the spinal defect either as a dermal substitute or beneath the skin closure, secured with sutures or bioadhesive.

Collectively, these results illustrate how tissue-engineered scaffolds are evolving from passive mechanical barriers to dynamic, bioactive platforms capable of orchestrating complex regenerative processes (Figure 5). Future directions include the integration of bioprinting, pre-vascularization, and smart materials with controlled release of growth factors with the goal to bridge the gap between preclinical experimentation and clinical translation.

Advances in 3D culture and bioprinting technologies are further accelerating this transition. Hydrogel constructs and printed neural patches now enable the recreation of key elements of spinal architecture, allowing the study of stem cell differentiation, axonal guidance, and scaffold–cell interactions in vitro. Complementary organoid systems are being developed to model patient-specific disease phenotypes and predict therapeutic responses, paving the way toward personalized prenatal regenerative strategies for myelomeningocele repair [46].

Recent work from our group has introduced a thermo-responsive Shape Memory Engineered Scaffold (SMES) specifically designed for minimally invasive fetal applications. The system is based on electrospun Polylactide-co-Polycaprolactone (PLA:PCL) nanofibers exhibiting a glass transition close to physiological temperature, enabling a one-way shape-memory effect triggered by mild heating. Through a controlled thermal cycle, the scaffold can be temporarily rolled into a compact tubular configuration and subsequently recover its original flat shape when exposed to 37 °C, allowing atraumatic unfolding directly over the spinal defect. Optimization of electrospinning parameters and shape-memory performance was achieved using a design-of-experiment approach, yielding scaffolds with high shape fixity (>88%) and recovery (>81%) before sterilization, and even higher recovery values following gamma irradiation sterilization due to reduced Tg and increased chain mobility [47]. Beyond their mechanical function, SMESs support cellularization: electrospun PLA:PCL nanofibers maintain structural integrity after shape-memory cycling, preserve biocompatibility, and sustain adhesion and viability of human dermal fibroblasts and AF-MSCs, making them suitable candidates for regenerative fetal repair. The shape-memory behavior and nanofibrous architecture of SMES, as characterized by Pisani et al., provide an initial proof-of-concept for its potential application in fetal MMC closure. Current evidence is limited to in vitro characterization of a single material composition, indicating feasibility for minimally invasive deployment and compatibility with bioactive interfaces, while highlighting the need for broader material optimization and in vivo assessment [48].

### 3.3. Molecular and Paracrine Modulation of Regeneration

Recent experimental studies highlight the pivotal role of molecular signaling and paracrine communication in driving tissue repair and neural regeneration after MMC repair. Rather than relying solely on cellular engraftment, these strategies exploit growth factors, signaling cascades, and secreted vesicles to activate endogenous repair mechanisms within the fetal microenvironment.

In preclinical models, controlled delivery of growth factors has proven effective in promoting epithelial and extracellular matrix (ECM) remodeling. Intra-amniotic alginate microparticles loaded with bFGF achieved partial epithelial closure in fetal rats, mainly through activation of the Mitogen-Activated Protein Kinase/Extracellular signal-Regulated Kinase (MAPK/ERK) and Phosphoinositide 3-Kinase/Protein Kinase B (PI3K/AKT) pathways, confirming the feasibility of in utero growth-factor therapy [28]. Similarly, modulation of the Sonic Hedgehog (SHH)–GLI1 axis has been shown to orchestrate epithelial–stromal interactions critical for both bladder and neural tube development; its targeted activation—potentially in coordination with Wnt/β-catenin signaling—may support regeneration of neural and urogenital tissues [39].

A complementary mechanism involves paracrine signaling by MSCs. UC-MSCs improve motor function and reduce fibrosis through PI3K/AKT, MAPK, and Transforming Growth Factor Beta (TGF-β) cascades [49]. BM-MSCs cultured on chitosan-gelatin scaffolds express Nestin and β-tubulin, markers of early neural differentiation, while releasing neurotrophic factors such as BDNF, nerve growth factor (NGF), and ciliar neurotrophic factor (CNTF). Among these, BDNF appears central, as UC-MSC therapy upregulates anti-apoptotic and anti-inflammatory mediators (BCL2, IL-4, IL-10) and downregulates CASP3, whereas CXCR4 overexpression enhances MSC migration and lesion homing, identifying a potential chemotactic therapeutic target [37].

Further evidence points to the regenerative potential of the MSC secretome and exosomes. P-MSCs exosomes carry proteins, lipids, and regulatory RNAs, including galectin-1 and neurotrophic mediators that activate PI3K/AKT, MAPK, Wnt, and focal adhesion signaling. Inhibition of galectin-1 abolishes these effects, nominating it as a promising pharmacologic target for neuroprotection [36]. Likewise, transamniotically delivered amniotic MSCs have been shown to home not only to spinal lesions but also to the fetal marrow and brain, suggesting systemic chemotactic and paracrine actions that extend beyond local tissue repair [50].

At the developmental level, regulators such as Lrp2 (megalin) coordinate apical constriction and planar cell polarity by controlling Vangl2 trafficking through Rab11 endosomes; its loss disturbs neuroepithelial organization and contributes to neural tube defects [11]. After MSC transplantation, upregulation of Brn3a and Runx1 indicates activation of sensory neurogenesis, where Brn3a supports dorsal root neuron survival and Runx1 governs neuronal subtype specification [38].

Taking together, these findings converge on a unified framework in which PI3K/AKT, MAPK, TGF-β, Wnt/β-catenin, SHH, and CXCR4 pathways act synergistically to mediate fetal tissue regeneration. The future of pharmacologic innovation in prenatal therapy will likely focus on precise modulation of these cascades through engineered biomaterials, targeted gene delivery, or small-molecule agonists, offering minimally invasive routes for in utero neuroprotection and functional restoration.

The main experimental strategies, models, and outcomes described above are synthesized in Table 1, which provides an integrated overview of current preclinical stem cell- and scaffold-based approaches for prenatal MMC repair.

## 4. Discussion

To place the available evidence into a translational readiness framework, the following discussion integrates preclinical and clinical data across experimental models, outcome measures, follow-up duration, reproducibility, and regulatory feasibility.

Despite remarkable progress in prenatal repair of MMC, the translation of innovative fetal and regenerative therapies remains constrained by major experimental, surgical, regulatory, and ethical barriers. Preclinical studies consistently demonstrate biological feasibility, but clinical readiness remains distant due to short follow-up, heterogeneity of methodologies, and lack of standardized evaluation metrics [28]. Even promising interventions such as intra-amniotic bFGF microparticles, UC-MSC-assisted repair, and BM-MSC-seeded scaffolds show partial benefits while suffering from variability in lesion size, high fetal loss, limited histologic or functional validation, and poor long-term data [37]. Large-animal ovine models provide improved anatomical relevance yet remain limited by postoperative spinal angulation, incomplete watertight closure, tethered cord risk, and primarily histologic outcome measures [45].

Surgically, fetoscopic approaches aim to reduce maternal morbidity compared with open fetal surgery, yet they remain technically demanding, marked by steep learning curves, equipment needs, and high PPROM rates (≈50–60%) across several series [30]. Inter-center variability in access type, CO_2_ insufflation, and closure technique complicates comparisons and hinders standardization [27]. Without large randomized controlled trials directly comparing open and fetoscopic repair, outcome interpretation remains limited. Specialized expertise, institutional resources, and consistent credentialing remain essential to ensure safety and reproducibility.

Regenerative strategies, including MSCs, exosomes, growth-factor delivery systems, and bioengineered scaffolds—have opened new avenues for enhancing neural preservation in utero. AF-MSCs and P-MSCs demonstrate paracrine neuroprotective, angiogenic, and anti-inflammatory effects, improving motor function and neuronal preservation in rodent and ovine models [36]. Scaffold-based approaches, including gelatin composites, collagen matrices, biocellulose membranes, fibrin patches, and aligned nanofibrous constructs, support epithelial coverage, reduce fibrosis, and guide axonal organization in various models [45]. More recently, SMES based on electrospun PLA:PCL have been developed to enable minimally invasive deployment and conformal coverage of the spinal defect, while maintaining nanofibrous architecture that supports cell adhesion and viability; however, current evidence remains largely limited to in vitro and early proof-of-concept studies [47]. Yet, biomechanical instability, scaffold detachment, contraction, and lack of long-term functional data remain recurrent obstacles. Even highly promising innovations—such as MSC-loaded fibrin patches in ovine MMC—show early neuroprotective signals but require extensive validation before translation [44].

Regulatory and manufacturing constraint further complicated progress. Cell-based or scaffold-associated therapies are classified as Advanced Therapy Medicinal Products (ATMPs), necessitating good manufacturing practice (GMP)-compliant production, potency assays, sterility testing, biodistribution analyses, and long-term evaluation of tumorigenicity and off-target migration—requirements that most early-phase groups cannot yet meet [46]. Exosome-based therapies face challenges regarding low yield, heterogeneity, and absence of standardized potency metrics [36]. TRASCET, although promising as a minimally invasive approach, still lacks large-animal validation and long-term safety datasets to satisfy regulatory expectations [51,53].

Ethical considerations remain central. Fetal interventions inherently involve balancing maternal risk with fetal benefit, raising questions of autonomy, non-directiveness, and equity. High maternal morbidity associated with some procedures and the absence of direct maternal benefit require rigorous consent processes and careful ethical oversight [52,54]. Broader issues of justice and access arise due to geographic concentration of expertise and resource requirements for fetal surgery and regenerative interventions.

Moving toward clinical translation will require harmonized multicenter pipelines integrating anatomically relevant large-animal models, standardized surgical techniques, robust functional endpoints, and long-term follow-up. Registry-based surveillance for neurological, orthopedic, and urological outcomes will be essential to establish durability and safety of both surgical and regenerative approaches. Randomized clinical trials comparing open and fetoscopic repair, as well as adjunctive MSC- or scaffold-based therapies, should incorporate uniform definitions for lesion closure, motor outcomes, shunt criteria, continence, and cognitive endpoints [46]. For cellular therapies, GMP-compliant manufacturing, optimized delivery platforms, and validated potency assays are fundamental prerequisites.

Ultimately, advancing MMC therapy from experimental innovation to reproducible clinical reality requires global collaboration, centralized training, harmonized ethical standards, and coordinated translational frameworks. Integrating surgical optimization with regenerative and molecular technologies may enable prenatal interventions that go beyond anatomical coverage toward preserving neurological function, continence, and long-term quality of life.

## 5. Conclusions and Future Perspectives

Prenatal repair of myelomeningocele has reshaped clinical practice, demonstrating that early surgical intervention can partially prevent secondary neural injury and improve motor and neurological outcomes. Despite these achievements, current techniques—whether open or fetoscopic—remain primarily protective rather than truly regenerative, and substantial functional limitations persist in many children. Recent advances in stem cell biology, bioengineered scaffolds, and minimally invasive delivery strategies highlight the emerging shift toward a regenerative paradigm. Placenta-derived MSCs, bioactive scaffolds, shape memory materials, and transamniotic cell-based approaches show encouraging neuroprotective and pro-regenerative potential in preclinical settings; however, their translation into clinical practice will require validation in large-animal models, standardized functional endpoints, and long-term safety data [55]. In this context, future studies would benefit from the adoption of a minimal standardized endpoint set, including clear and harmonized definitions of lesion closure and dural integrity at birth, standardized motor function assessments, uniform criteria for cerebrospinal fluid diversion or shunt placement, clinically relevant bladder and bowel continence outcomes, and age-appropriate neurocognitive developmental endpoints, to improve comparability and translational relevance across centers. Moving forward, progress will depend on the standardization of manufacturing processes for cell-based products, optimization of delivery platforms, and robust long-term assessment in relevant large-animal models and early human studies. Convergence between surgical refinement, regenerative medicine, and molecular technologies may ultimately enable prenatal interventions designed not only to protect neural tissue but to actively restore function. Achieving this goal will require coordinated multicenter research efforts, structured registries, and ethical frameworks that ensure both maternal safety and equitable access. By integrating these innovations, prenatal therapy may evolve toward interventions capable of improving long-term neurological function and quality of life for future generations of children with spina bifida.

## Figures and Tables

**Table 1:** Overview of in vivo and in vitro studies on stem cell- and scaffold-based strategies for fetal repair of myelomeningocele. The table summarizes key experimental parameters, outcomes, and limitations across various biomaterial and cellular platforms, highlighting translational progress toward regenerative in utero therapy.

MSCs Source	Scaffold Type	Surgery Type	Application	Effect *	Limitation	Ref.
N/A	Alginate microparticles (MPs) + bFGF	Minimally invasive intraamniotic injection	Rat model of MMC	Partial (~30%) skin-like coverage; specific binding of alginate MPs to defect	Small, variable sample; subjective closure; long-term safety unverified	[9]
AF-MSCs	Gelatin hydrogel composites (sponges, microspheres, sheets)	Open fetal surgery	Retinoic acid (RA)-induced MMC rat model	Enhanced cell ingrowth and epidermal coverage; coatings ↑ AF cell adhesion	Short gestation; no postnatal follow-up; poor microsphere retention	[10]
Syngeneic EGFP-labeled BM-MSCs	N/A	Intra-amniotic BM-MSC injection	Rat model OSB	Improved AF environment; ↑ neurotrophic cytokines; ↓ apoptosis; ↑ synaptic development	Partial repair; weak Activin A effect; limited validation and ethical constraints	[15]
N/A	Gelatin/collagen sponges + bFGF	Surgical repair of MMC	Fetal sheep model of surgically created MMC	Complete defect coverage; bFGF ↑ granulation and epithelialization; partial Hindbrain Herniation (HH) reversal	Small, variable sample; scaffold detachment; no neuro tests; tethering risk	[32]
UC-MSCs	Fibrin patch	Prenatal surgical repair of MMC	Ovine (fetal lamb) MMC model	↑ Motor function and neuron density; ↓ fibrosis (paracrine); no incontinence or tumors	Small, variable sample; scaffold detachment; no neuro tests; tethering risk	[26]
N/A	Bovine decellularized collagen dural patches (DuraGen^®^, Durepair™)	Immediate OSB repair with dural patch and skin closure	Fetal rabbit model of surgically induced OSB	Preserved motor neurons; ↓ inflammation; no CSF leak → prevented HH	Rabbit model limits relevance; high mortality; small sample; short follow-up	[33]
P-MSCs and AF-MSCs	N/A	TRASCET	Rodent model of experimental spina bifida	Similar partial/complete coverage with AF-MSCs (46%) and P-MSCs (47%)	Rodent model; poor cell tracking; no significant improvement; translational limits	[35]
MSCs and CD34^+^ HSPCs from SB pediatric patients	POC elastomeric scaffold	Augmentation surgery (partial bladder cystectomy)	Nude rat urinary bladder augmentation model	MSC/CD34^+^ HSPC co-transplantation ↑ bladder regeneration, vascularization, and nerve growth	Mechanisms unclear; pediatric applications underexplored	[39]
BM-MSCs	Chitosan-gelatin scaffold porosity >90% interconnected pores (100–400 um)	Intrauterine transplantation of the BM-MSC-scaffold construct	Rat model of OSB	↓ Spinal defect; near-complete skin closure; BM-MSCs → neural stem/neuronal phenotypes	Low cell survival; early-stage approach; long-term and ethical validation needed	[37]
P-MSCs	N/A	In vitro SH-SY5Y apoptosis model	SH-SY5Y neuroblastoma cell apoptosis model	P-MSC exosomes → neuroprotection via galectin-1; ↓ apoptosis; ↑ neurite outgrowth	Low exosome yield; limited in vivo relevance; single dose and isolation constraints	[36]
AF-MSCs	N/A	TRASCET, involving intra-amniotic injections	Rodent model of experimental spina bifida (induced by RA)	↑ Tissue regeneration and partial skin coverage; AF-MSCs ≈ P-MSCs; ↓ Chiari II via paracrine effects	Experimental; needs long-term validation; poor cell tracking; ethical/regulatory concerns	[51]
BM-MSCs	N/A	In utero MSC transplantation (fetal surgery/microinjection)	Rat model of OSB	↑ Sensory neuron differentiation/protection; ↑ Brn3a^+^, Runx1 in spinal cord and DRG	Low MSC differentiation; functional maturity and relevance unconfirmed	[38]
Autologous fetal fibroblasts and keratinocytes (from skin biopsy)	fDESS built on Collagen-based hydrogels	In utero transplantation (open fetal surgery) for primary skin closure in SB repair	Fetal sheep model	Successful in utero autologous skin graft; near-normal epidermis and strong neovascularization	Risk of infection and graft contraction; required open fetal surgery, limiting clinical applicability	[40]
Ovine fetal chondrocytes (knee cartilage–derived)	Collagen I hydrogels (compressed/non-compressed) ± fibrin glue	In utero surgical spina bifida repair (Target goal); In vitro study only	Ovine fetal chondrocytes in 3D scaffold-assisted culture	Fetal chondrocytes retained chondrogenic potential; collagen I scaffolds supported cartilage-like tissue	Collagen contraction; fibrin glue ineffective; need stable bioprintable materials	[41]
N/A	Collagen-based hydrogels for fDESS (fetal dermo-epidermal skin substitutes)	In utero transplantation (Open fetal surgery onto excisional wounds)	Fetal sheep model	Successful in utero autologous skin substitute; regenerated normal epidermis with confirmed origin and rich vascularization	Infection risk and graft shrinkage; required open fetal surgery, limiting translational feasibility	[42]
N/A	BCF (Cellulose Solution, LLC)	Hysterotomy (Patch secured with mattress suture)	RA-induced physiologic SB rat model (repair E20, eval. 30–34 h)	Complete absence of cellular migration around or within the patch. No MPO- or CD3-positive cells inside the patch	BCF patches lacked mechanical strength; induced macrophage infiltration and inflammation, potentially leading to fibrosis	[43]
Ovine Umbilical cord-MScs	Fibrin patch (EVICEL^®^ kit fibrinogen/thrombin- Ethicon, Raritan, NJ, USA)	In utero MMC repair with patch placement and skin closure	Ovine MMC model	Partial motor/urinary improvement; ↑ neuronal density, gray matter; ↓ fibrosis; complete dural healing; no tumors	Limited motor gain; nonsignificant SLR; surgical variability; early assessment bias	[44]
N/A	Aligned biodegradable Nanofibrous Scaffolds (NSs); Made of PLLA	Open fetal MMC repair with intra- and extradural scaffold placement	Sheep Model (Fetal Lambs); Surgically induced MMC defect	Feasible and safe; integrated scaffold without inflammation or foreign-body reaction	Pilot study (n = 2); further studies needed for neural integration, cell delivery, and functional recovery	[45]
AF-MSCs	Shape Memory Engineered Scaffold (SMES)	N/A	In vitro evaluation on NHDF: shape-memory performance confirmed; supports cell adhesion and viability	High surface area and porosity; stimulus-responsive shape-memory effect; tunable shape fixity and recovery; supports cell adhesion and alignment; enables minimally invasive deployment	Only one copolymer composition was tested. It needs validation.	[47]
P-MSCs	Durepair (cadaveric dura) Commercially available patch supplemented with stem cells.	Open Fetal Surgery	Human	First-in-human fetal MMC repair with P-MSC-ECM graft (safety + preliminary efficacy).	Not personalized medical device and remains invasive procedure	CuRe Trial (NCT04652908) [52]

* The rows indicate:↑ increase; ↓ decrease; → denotes outcome or direction of effect.

## Data Availability

No new data were created or analyzed in this study.

## Abbreviations

The following abbreviations are used in this manuscript:

AChE	Acetylcholinesterase
AFP	Alpha-Fetoprotein
AKT	Protein Kinase B
ATMP	Advanced Therapy Medicinal Product
bFGF	Basic Fibroblast Growth Factor
BCF	Biocellulose Film
BCL2	B-Cell Lymphoma 2
BDNF	Brain-Derived Neurotrophic Factor
BM-MSCs	Bone Marrow-Derived Mesenchymal Stem Cells
CNS	Central Nervous System
CNTF	Ciliary Neurotrophic Factor
CPC	Choroid Plexus Cauterization
CO_2_	Carbon Dioxide
CSB	Closed Spina Bifida
CSF	Cerebrospinal Fluid
CuRe	Cellular Therapy for In Utero Repair of Myelomeningocele Trial
DRG	Dorsal Root Ganglion
ECM	Extracellular Matrix
EGFP	Enhanced Green Fluorescent Protein
ERK	Extracellular signal-Regulated Kinase
ETV	Endoscopic Third Ventriculostomy
fDESS	Fetal Dermo-Epidermal Skin Substitute
FGF2	Fibroblast Growth Factor 2
GMP	Good Manufacturing Practice
HH	Hindbrain Herniation
HSPCs	Hematopoietic Stem/Progenitor Cells
HUC	Human Umbilical Cord
iPSCs	Induced Pluripotent Stem Cells
Lrp2	Low-Density Lipoprotein Receptor-Related Protein 2 (Megalin)
MAPK	Mitogen-Activated Protein Kinase
MMC	Myelomeningocele
MoM	Multiples of the Median
MOMS	Management of Myelomeningocele Study
MPs	Microparticles
MRI	Magnetic Resonance Imaging
MSC/MSCs	Mesenchymal Stem Cell(s)
MSAFP	Maternal Serum Alpha-Fetoprotein
NGF	Nerve Growth Factor
NT	Neural Tube
NTD/NTDs	Neural Tube Defect(s)
NSs	Nanofibrous Scaffolds
OSB	Open Spina Bifida
PCL	Polycaprolactone
PCP	Planar Cell Polarity
PML	Percutaneous–Mini-Laparotomy
P-MSCs	Placental Mesenchymal Stromal Cells
PI3K	Phosphoinositide 3-Kinase
PLA	Polylactic Acid
PLLA	Poly-L-Lactic Acid
POC	Poly(1,8-Octanediol-co-Citrate)
PPROM	Preterm Premature Rupture of Membranes
QoL	Quality of Life
RA	Retinoic Acid
SB	Spina Bifida
SMES	Shape Memory Engineered Scaffold
SHH	Sonic Hedgehog
SIS	Small Intestinal Submucosa
TGF-β	Transforming Growth Factor Beta
TRASCET	Transamniotic Stem Cell Therapy
UC-MSCs	Umbilical Cord-Derived Mesenchymal Stem Cells
USD	United States Dollars
VEGF	Vascular Endothelial Growth Factor
VP	Ventriculoperitoneal
WJ-MSCs	Wharton’s Jelly-Derived Mesenchymal Stem Cells
Wnt	Wingless-Related Integration Site Signaling Pathway
